# Progress in the Prevention of Mother to Child Transmission of HIV in Three Regions of Tanzania: A Retrospective Analysis

**DOI:** 10.1371/journal.pone.0088679

**Published:** 2014-02-13

**Authors:** Ann M. Buchanan, Dorothy E. Dow, Charles G. Massambu, Balthazar Nyombi, Aisa Shayo, Rahma Musoke, Sheng Feng, John A. Bartlett, Coleen K. Cunningham, Werner Schimana

**Affiliations:** 1 Kilimanjaro Christian Medical Centre, Moshi, Tanzania; 2 Division of Infectious Diseases, Department of Pediatrics, Duke University Medical Center, Durham, North Carolina, United States of America; 3 Duke Global Health Institute, Duke University, Durham, North Carolina, United States of America; 4 Ministry of Health and Social Welfare, Dar es Salaam, Tanzania; 5 Kilimanjaro Christian Medical Centre Clinical Laboratory, Moshi, Tanzania; 6 Department of Biostatistics and Bioinformatics, Duke University, Durham, North Carolina, United States of America; 7 Division of Infectious Diseases, Department of Medicine, Duke University Medical Center, Durham, North Carolina, United States of America; 8 Elizabeth Glaser Pediatric AIDS Foundation, Dar es Salaam, Tanzania; University of British Columbia, Canada

## Abstract

**Background:**

Mother to child transmission (MTCT) of HIV-1 remains an important problem in sub-Saharan Africa where most new pediatric HIV-1 infections occur. Early infant diagnosis of HIV-1 using dried blood spot (DBS) PCR among exposed infants provides an opportunity to assess current MTCT rates.

**Methods:**

We conducted a retrospective data analysis on mother-infant pairs from all PMTCT programs in three regions of northern Tanzania to determine MTCT rates from 2008–2010. Records of 3,016 mother-infant pairs were assessed to determine early transmission among HIV-exposed infants in the first 75 days of life.

**Results:**

Of 2,266 evaluable infants in our cohort, 143 had a positive DBS PCR result at ≤75 days of life, for an overall transmission rate of 6.3%. Transmission decreased substantially over the period of study as more effective regimens became available. Transmission rates were tightly correlated to maternal regimen: 14.9% (9.5, 20.3) of infants became infected when women received no therapy; 8.8% (6.9, 10.7) and 3.6% (2.4, 4.8) became infected when women received single-dose nevirapine (sdNVP) or combination prophylaxis, respectively; the lowest MTCT rates occurred when women were on HAART, with 2.1% transmission (0.3, 3.9). Treatment regimens changed dramatically over the study period, with an increase in combination prophylaxis and a decrease in the use of sdNVP. Uptake of DBS PCR more than tripled over the period of study for the three regions surveyed.

**Conclusions:**

Our study demonstrates significant reductions in MTCT of HIV-1 in three regions of Tanzania coincident with increased use of more effective PMTCT interventions. The changes we demonstrate for the period of 2008–2010 occurred prior to major changes in WHO PMTCT guidelines.

## Introduction

More than 90% of the world’s HIV-infected children reside in sub-Saharan Africa, where mother to child transmission (MTCT) of human immunodeficiency virus type 1 (HIV-1) remains the most important cause of pediatric HIV infection [1]. Furthermore, 50% of HIV-infected children will die by the age of two years without proper care and treatment [2]. Recent advances in prevention measures have resulted in vertical transmission rates in industrialized countries of less than 2% [3]–[5], and MTCT rates have decreased considerably in many resource-limited areas as well [6]–[10]. The international community has responded to the continued threat of MTCT of HIV-1 with the development of the Global Plan for elimination of MTCT by 2015 put forth by UNAIDS [11].

Tanzania is one of the 22 countries with the highest estimated numbers of pregnant women living with HIV, and as such is a focus country for MTCT elimination [11]. HIV prevalence among pregnant women is 6.8%, compared to an overall population prevalence of 5.7% [12]. As in other sub-Saharan African countries, tremendous progress has been made in Tanzania with regard to accessibility and uptake of prevention of mother to child transmission (PMTCT) services. Tanzanian PMTCT program data assessments for 2010 estimate that of the 119,000 HIV-infected women who give birth annually, approximately 70% receive some form of antiretroviral treatment for PMTCT [13]. Programs, however, vary widely, both across and within regions, and medications available to women and their infants vary as well.

Prior to 2011, HIV-infected pregnant women were eligible for highly active antiretroviral therapy (HAART) only if they had World Health Organization (WHO) Stage IV disease, or WHO Stage III disease *and* a CD4 count below 350 cells/uL. Pregnant women with stage I/II disease were eligible for HAART only when their CD4 count fell below 200 cells/uL. Women who did not qualify for HAART were eligible to start zidovudine (AZT) prophylaxis beginning at 28 weeks, along with single-dose nevirapine (sdNVP) and lamivudine (3TC) at labor/delivery and postpartum but where this was neither available nor feasible, single-dose nevirapine (sdNVP) alone was recommended. Exposed newborns were eligible to receive sdNVP within 72 hours of life and twice daily AZT for one week, or 4 weeks if the mother had received less than 4 weeks of prophylaxis; the use of daily nevirapine for breastfeeding infants had not yet been implemented.

In recent years, access to HIV early infant diagnosis (EID) has also increased in Tanzania. Prior to 2008 the country relied on antibody testing for infant diagnosis, making the diagnosis challenging due to the presence of maternal antibody up to 12–18 months of life. The country’s current commitment to the provision of EID services using PCR on dried blood spots (DBS) in the first eight weeks of life not only allows for early diagnosis and treatment of infected infants, but can also be used as a tool to measure the effectiveness of PMTCT programs.

In order to determine uptake of PMTCT treatment regimens, prevalence of MTCT, and the relationship between these, we reviewed DBS PCR results from HIV-exposed infants, combined with PMTCT treatment information collected from national registries at all health facilities providing PMTCT services in three regions of northern Tanzania.

## Methods

### Design, Setting, and Data Collection

This study is a retrospective data analysis on mother-infant pairs from PMTCT program records in three regions of Tanzania, using stored DBS samples stored as standard of care from HIV-exposed infants to determine early MTCT prevalence rates from HIV-exposed children ≤75 days of age. “Early” MTCT in this context, therefore, encompasses in-utero transmission, intrapartum transmission, and early breast milk infection. This study was conceived and designed in conjunction with members from the Tanzanian Ministry of Health and Social Welfare Laboratory Services Division. Following Tanzanian national guidelines, HIV-exposed infants undergo DBS PCR testing at their first visit to a Reproductive Child Health (RCH) clinic, usually between 4–8 weeks of age. Using a heel prick, five circles are filled with blood on a specific filter paper (Whatman) and sent to a Zonal PCR Laboratory. The Kilimanjaro Christian Medical Centre Clinical Laboratory is one of four such laboratories in Tanzania responsible for processing, testing, and storing DBS results for the three regions we studied. In the laboratory, one DBS circle is used to run a DNA-PCR test and if positive, a second circle is analyzed to confirm the first result. Only if both PCR tests are positive does the result become classified as positive and this result is then sent back to the RCH clinic.

Every DBS card is labelled with the infant’s name and a unique identifier which is also recorded in the EID and PMTCT Mother-Child Follow-up Register that remains at the clinical site. Other information recorded in the registry includes: date of birth, date sample taken, PMTCT regimen used by the mother, infant regimen, infant feeding option, and initiation of co-trimoxazole prophylactic therapy.

Three research assistants, each assigned to one region of northern Tanzania (Arusha, Kilimanjaro, and Tanga) visited all health facilities providing PMTCT and EID services within the regions. Using these national registries, de-identified information was collected from all mother-infant pairs where the infant received a first DBS PCR between January 1, 2008 and September 30, 2010. During this time period, possible maternal PMTCT regimens included either: 1) no medication; 2) sdNVP only; 3) combination prophylaxis (AZT, recorded as>or <4 weeks prior to delivery, and sdNVP and lamivudine (3TC) given at labor and delivery along with AZT plus 3TC for one week); or 4) HAART. The infant regimens provided during this time period included sdNVP at birth, with or without AZT (either for 1 week or 4 weeks depending on duration of maternal prophylaxis). After reviewing the registries of all facilities for the three regions as described, all positive DBS PCR results recorded at site registries were cross-checked by retrieving the original samples from the zonal laboratory (Kilimanjaro Christian Medical Centre). This enabled us to exclude any potential false positive PMTCT transcription errors from site PMTCT registries.

### Statistical Analysis

Data were entered using the Cardiff Teleform system (Cardiff Inc., Vista, CA, USA) into an Access database (Microsoft Corp., Redmond, WA, USA). All data were manually reentered into a second Microsoft Access database and compared using Stata version 12 (StataCorp LP, College Station, TX, USA). All subsequent analyses were performed with Stata version 12, using a 5% level of significance (two-sided). Descriptive statistics were used to summarize demographic data. Categorical data were compared using the Chi-square test or Fisher’s exact test, where appropriate.

### Ethical Statement

The study was approved by the Duke University Institutional Review Board, the KCMC Research Ethics Committee, and the National Institute of Medical Research in Tanzania. All data collection was retrospective in nature and was collected as part of the routine delivery of PMTCT services in Tanzania. All ethical bodies approved the request for a waiver of informed consent due to the fact that all information collected was on de-identified patient information and PCR samples.

## Results

**Figure 1:**
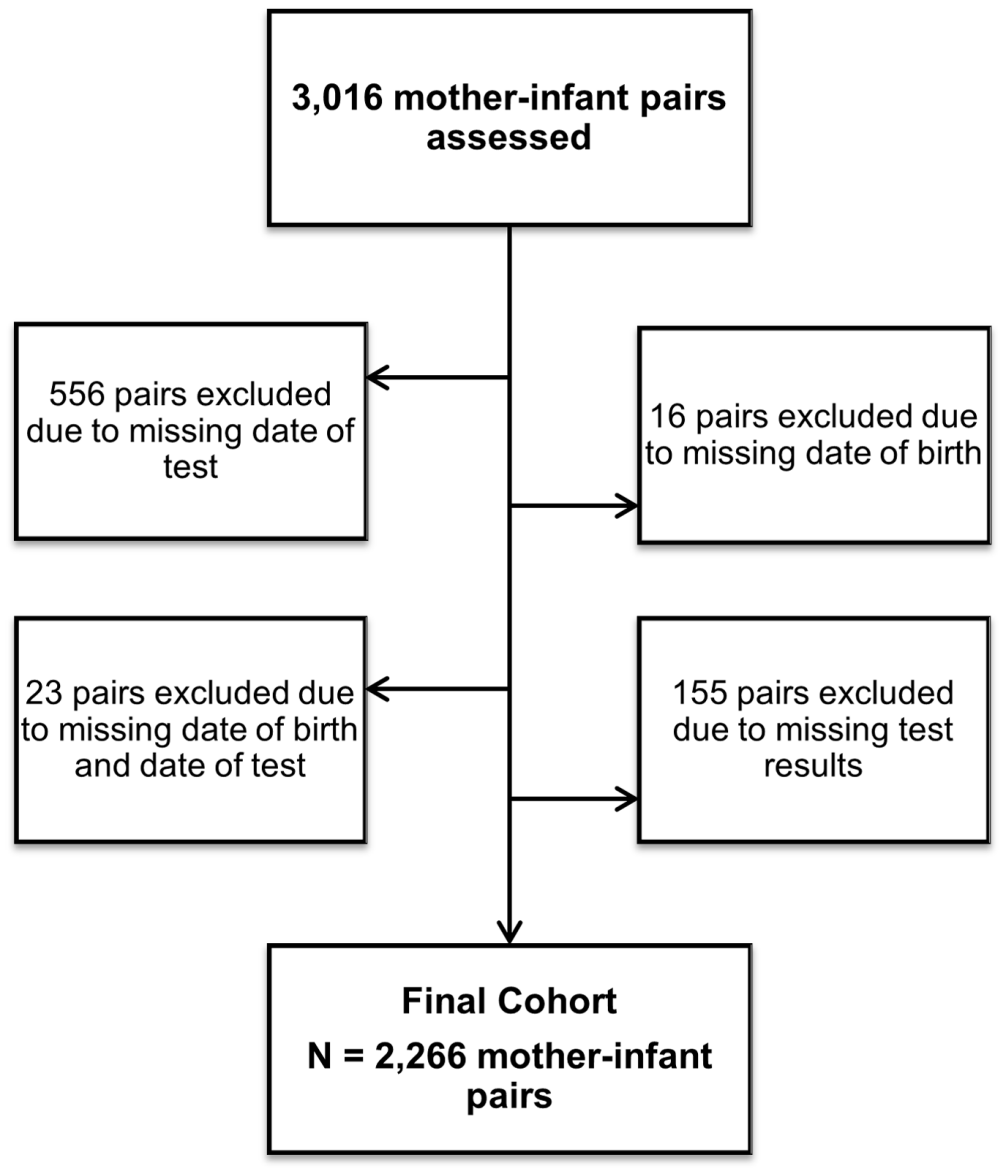
Population Flowchart.

Data from 3,016 mother-infant pairs were collected from 98 health facilities representing a total of 22 districts in the Arusha, Kilimanjaro, and Tanga Regions. Of these, 595 pairs were excluded due to a missing date of birth and/or date of test information, and 155 pairs were excluded due to missing test results (Figure 1). In total, data from 2,266 mother-infant pairs were used in the final analysis. The mean age of first infant PCR was 40 days, standard deviation (SD) 13. The median (range) maternal age at time of delivery was 28 years (16, 46), and 86.5% of infants were reported as exclusively breastfeeding at the time of first DBS PCR.

During the study period, 7.4% of mothers received no medication, 36.1% received sdNVP only, 42.6% received combination prophylaxis, 10.7% received HAART, and 3.2% had no antiretroviral information recorded. Among infants, 9.0% received no medication, more than one-third (35.2%) received sdNVP only, and the majority (50.8%) received sdNVP with 1–4 weeks of AZT prophylaxis. An additional 5.0% had no information recorded.

**Figure 2:**
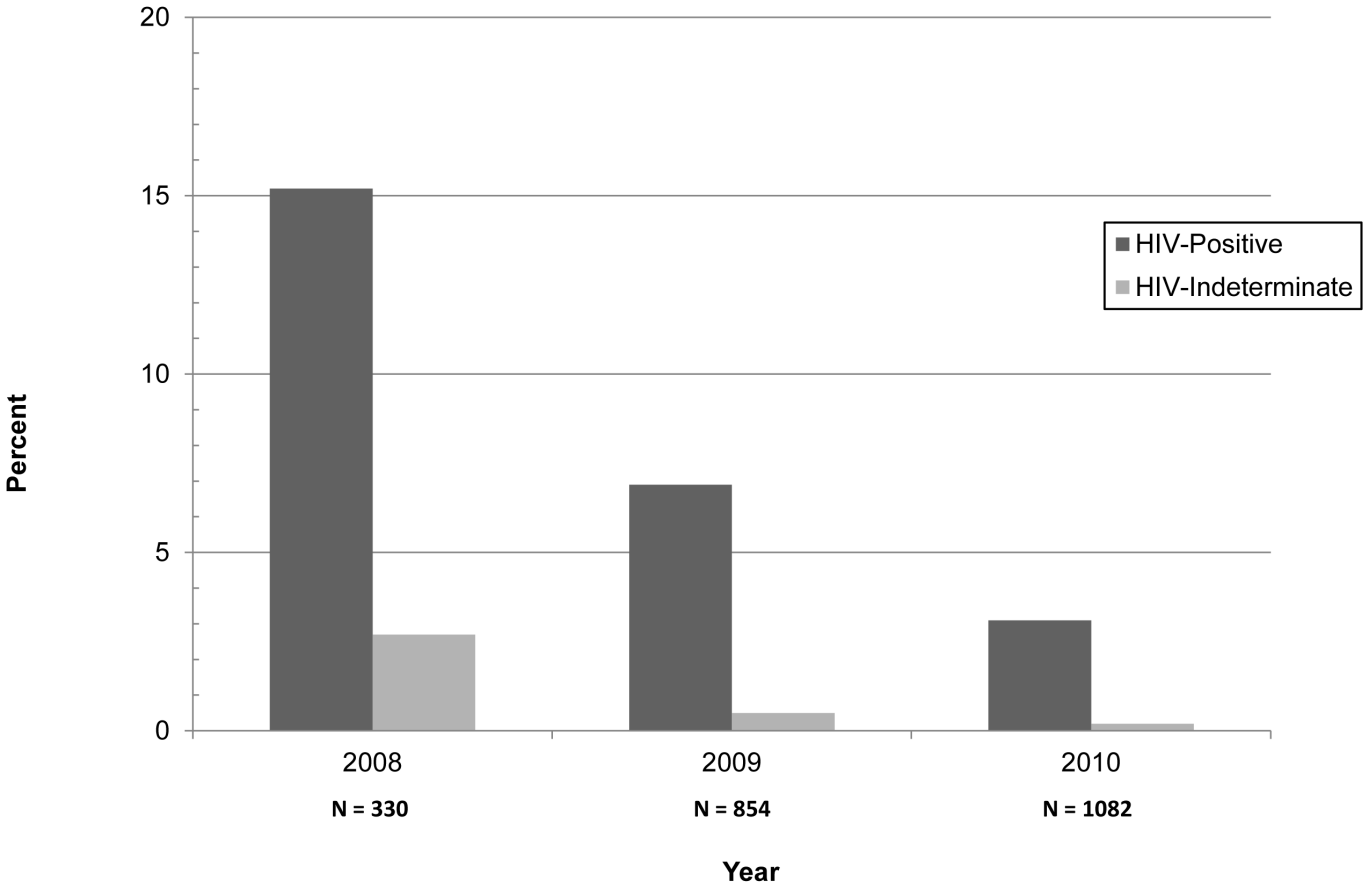
Positive Infant Dried Blood Spot PCR by Year for Arusha, Kilimanjaro, Tanga Regions.

Of the 2,266 infants in our cohort, a total of 143 tested positive on DBS PCR in the first 75 days of life, for an overall HIV-1 MTCT prevalence of 6.3% (95% CI: 5.3%, 7.3%). Transmission decreased substantially over the period of study, as more effective regimens were rolled out. Notably, uptake of DBS PCR testing more than tripled over the course of the study period, from 330 infants tested in 2008 to 1082 tested in the first 9 months of 2010 (Figure 2). Transmission rates were tightly correlated to maternal regimen; 14.9% (9.5%, 20.3%) of infants became infected when women received no therapy, compared to 8.8% (6.9%, 10.7%) and 3.6% (2.4%, 4.8%) when women received sdNVP or combination prophylaxis, respectively. The lowest MTCT rates occurred when women received HAART, with 2.1% transmission (0.3%, 3.9%) (Table 1). Women who received sdNVP only had a 41% decreased risk of MTCT (38%, 90%, p = 0.01) compared to women who received no antiretrovirals. Women who received combination prophylaxis had a 59% decreased risk of transmission compared to women receiving sdNVP alone (28%, 61%, p<0.01).

**Table 1:** Rates of Infant DBS PCR Results by 75 Days of Age According to Antiretroviral Regimen Received for Arusha, Kilimanjaro, Tanga Regions.

Maternal ARVRegimen (n)	Infant DBS PCRPositive (95% CI)*	Infant ARV Regimen				
		None	sdNVP	NVP+AZT	Not Recorded	Total
**None (168)**	**14.9%**	**17.6%**	**14.0%**	**7.7%**	**25%**	**168**
	(9.5, 20.3)	(8.9, 26.3)	(3.6, 24.4)	n = 39	n = 12	
		n = 74	n = 43			
**sdNVP (818)**	**8.8%**	**11.1%**	**10.1%**	**4.9%**	**4.3%**	**818**
	(6.9, 10.7)	(3.3, 18.9)	(7.6, 12.6)	(1.6, 8.2)	n = 47	
		n = 63	n = 546	n = 162		
**Combination** **Prophylaxis (966)**	**3.6%**	**5.4%**	**5.5%**	**3.2%**	**3.6%**	**966**
	(2.4, 4.8)	n = 37	(1.6, 9.5)	(2.0, 4.4)	n = 28	
			n = 128	n = 773		
**HAART (243)**	**2.1%**	**0%**	**1.8%**	**2.6%**	**0%**	**243**
	(0.3, 3.9)	n = 22	n = 55	n = 156	n = 10	
**Other/Not** **Recorded (71)**	**8.7%**	**12.5%**	**11.5%**	**0%**	**12.5%**	**71**
	(2.1, 15.3)	n = 8	n = 26	n = 21	n = 16	
**Total (2,266)**	**6.3%**	N = 204	N = 798	N = 1,151	N = 113	**2,266**
	(5.3, 7.3)					

*Where applicable.

**Figure 3:**
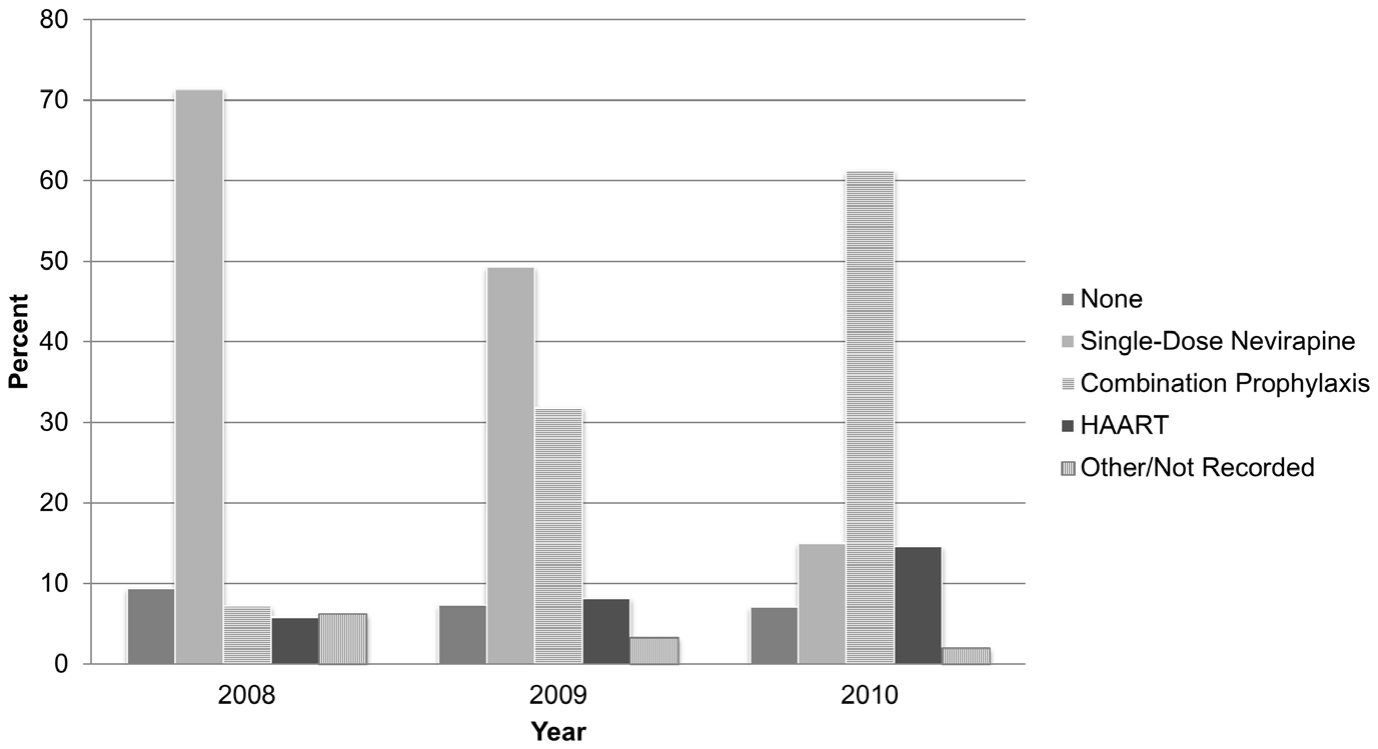
Maternal Antiretroviral Regimen by Year, Arusha, Kilimanjaro, Tanga Regions.

Treatment regimens changed dramatically over the 33-month period of study. In 2008, 72.4% (67.6%, 77.2%) of women in the cohort received sdNVP only, decreasing to 14.6% (12.5%, 16.7%) in 2010. Conversely, just 7% (4.2%, 9.8%) of women received combination prophylaxis in 2008, increasing to 61.8% in 2010 (58.9%, 64.7%). Only 243 women received HAART although the proportion did increase over time with 5.5% of the cohort receiving HAART in 2008 and 14.4% in 2010 (Figure 3).

In addition to maternal prophylaxis, infant prophylaxis also plays a critical role in PMTCT. In this cohort, 11.3% (7%, 15.6%) of the 204 infants who received no therapy had a positive DBS PCR, compared to 9% (7%, 11%) of the 798 babies who received sdNVP. Over half of infants in the cohort received a combination of zidovudine and nevirapine, and this group represented the lowest MTCT transmission rate at 3.5% (2.4%, 4.6%) (Table 1).

Finally, analyzing mother and infant regimens together we see that when neither mother nor infant received any intervention, the transmission rate was 17.6% (8.9%, 26.3%). However, when the baby did receive prophylaxis in the form of sdNVP, transmission, as measured by the first DNA PCR, was reduced by more than 20% (p = 0.61). Table 1 shows results for each possible combination of maternal and infant regimens. The majority of these combinations result in small numbers and p values are not significant. One exception, however, is comparing mothers and infants who received sdNVP only to mothers receiving sdNVP with infants receiving NVP+AZT. Here we see a 51% reduction in transmission from 10.1% to 4.9% (p = 0.049).

## Discussion

These data, representing a comprehensive analysis from PMTCT programs providing EID services in three regions of northern Tanzania, show that MTCT has declined from 15.2% in 2008 to 3.1% in 2010, with an overall prevalence of 6.3% for this 33-month period. The change over time from primarily sdNVP use to a predominance of combination prophylaxis, combined with the dramatic increase in use of DBS PCR for EID, is a remarkable demonstration of PMTCT implementation and effective coordination of services. Tanzania has shown great success since its commencement of PMTCT services in 2000, with the development of newer guidelines and effective health policy implementation. By 2010, an estimated 94% of all Reproductive and Child Health facilities across the country were providing PMTCT services [13].

The revised Tanzanian HIV clinical guidelines introduced on a national level in 2011 include starting AZT prophylaxis twice daily beginning at 14 weeks gestation for HIV-infected pregnant women, with nevirapine and 3TC given peripartum for mothers who receive <4 weeks of AZT prior to delivery; or lifelong HAART for women with WHO Stage III or IV disease and/or an absolute CD4 count below 350 cells/µL [14]. Infants receive daily nevirapine for at least the first 6 weeks of life, and this continues until one week after complete breastfeeding cessation if the mother herself is not on HAART [14]. As new evidence on best PMTCT practices become available, guidelines continue to change. At present, Tanzania is rolling out the WHO B+ Option (which provides triple anti-retroviral therapy to all HIV-infected pregnant or breastfeeding women regardless of clinical stage or CD4 count – for life). Thus, the development and implementation of new guidelines demonstrate tremendous progress in the field of PMTCT in Tanzania; however, it is well known that the successful execution of services and the ability to reach all infected women and their infants takes time and will vary according to location and healthcare setting.

For our study, we found an MTCT rate of 14.9% when the mother received no ARV intervention. The MTCT rates we observed when no maternal or infant prophylaxis was reported (17.6%) are slightly lower than those of recently published clinical trials data, though direct comparisons are difficult. Among non-breastfeeding populations, the risk of MTCT of HIV-1 ranges from 15–30% when there is no maternal or infant ARV prophylaxis. Breastfeeding, however, increases the risk of transmission by anywhere from 5–20%, resulting in overall transmission rates of 20–45% [15]. Furthermore, non-exclusive breastfeeding is a well-established factor increasing transmission risk [16], [17] compared with exclusive breastfeeding and this further complicates comparisons. More than 85% of the population we studied was reported to be exclusively breastfed at the time of the infant’s first DBS PCR. The PETRA trial conducted in Tanzania, South Africa, and Uganda showed a transmission rate of 15.3% at 6 weeks of life among breastfed infants when no regimen was given to mother or infant – slightly lower than our cohort though ours also encompassed infants beyond the age of six weeks. [18] Recent studies conducted in Nigeria and Zambia showed higher transmission rates, of 19.5% and 21.8%, respectively, when there was no maternal or infant regimen given [19], [20]; yet again, these were for infants up to the age of 6 weeks only.

Women who received combination prophylaxis had low HIV-1 transmission rates as would be expected, with an overall transmission rate of 3.6%. Clinical trials results have demonstrated transmission rates ranging from 2–9%, depending on length of regimen and whether or not the infant breastfed [18], [21], [22]. At the time our study cohort received PMTCT care, guidelines recommended AZT prophylaxis beginning at 28 weeks gestation for mothers who did not qualify for HAART. However, the women in our combination prophylaxis category included those who started as early as 28 weeks, as well as those who started just prior to delivery; thus, again, what we have observed in our field studies does seem to be lower than clinical trials results.

The number of women on HAART during the study period comprised only a small proportion of the entire cohort, making comparisons with this group difficult due to small sample size. Our MTCT rate observed of 2.1% among women on HAART is in line with other studies from developed countries, where maternal HAART among non-breastfed populations reduces MTCT to 2% or less [23]–[26]. In this analysis women receiving HAART actually comprise a group with higher transmission risk as it only includes women with WHO Stage IV disease, WHO Stage III disease *and* CD4<350 cells/ul, or a CD4 count<200 cells/uL regardless of WHO stage.

Finally, as a post-hoc analysis we evaluated the added benefit of infant AZT to reduce HIV transmission as compared to mother-infant pairs who received sdNVP only. The statistically significant reduction in transmission by 51% is interesting, but requires further study with a larger cohort.

**Figure 4:**
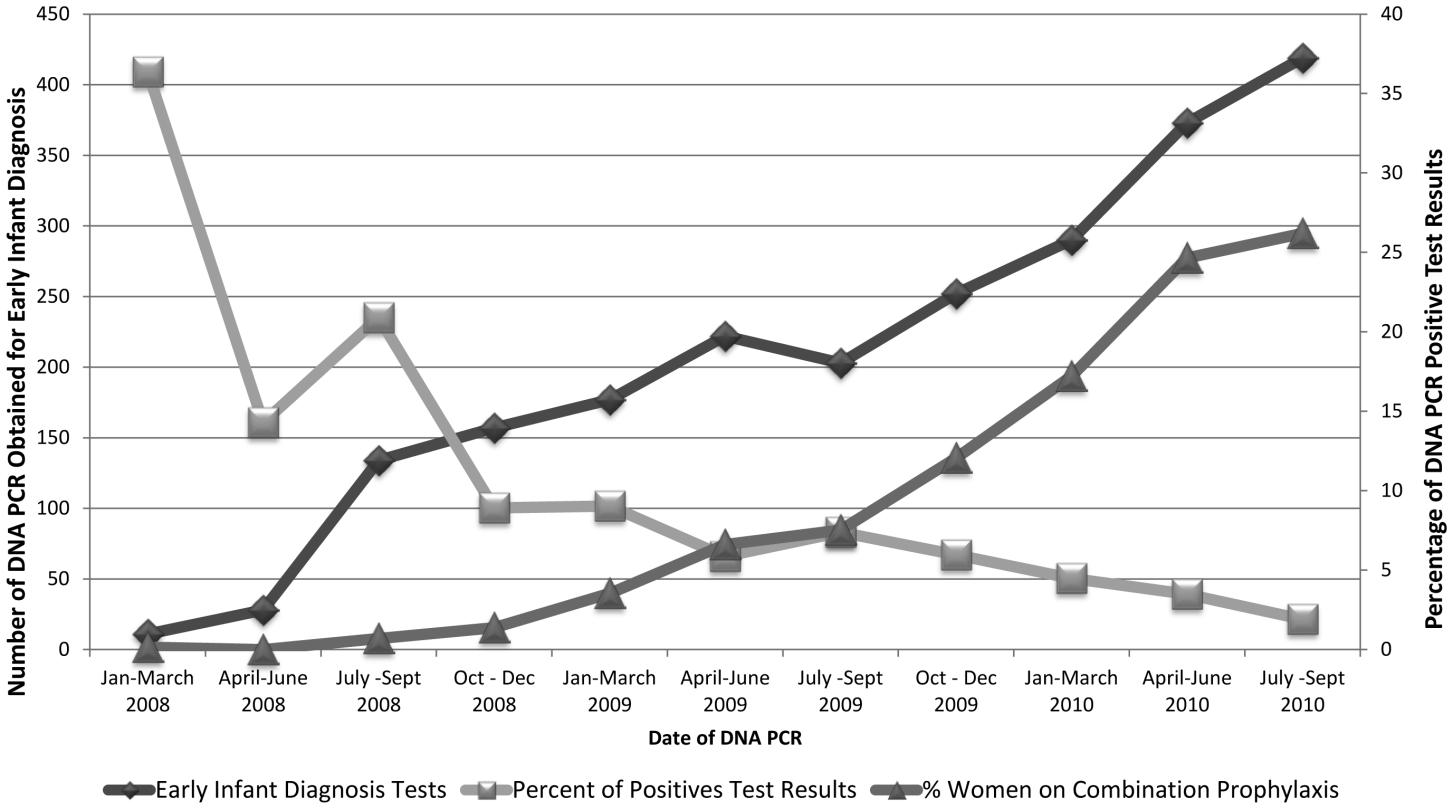
Changes in Early Infant Diagnosis Scale-Up and Percent HIV DNA PCR Positive over Study Period, January 2008– September 2010.

With changing national guidelines we demonstrated a shift in maternal treatment regimens over time toward more women receiving combination prophylaxis and fewer receiving sdNVP (Figures 3 & 4). The decrease in transmission rates for this time period shows real progress for Tanzania (Figure 4). Furthermore, the number of women on HAART from 2008 to 2010 more than doubled. Perhaps most striking, the number of infants being tested for this time period, shortly after EID rollout began, more than tripled over the 33-months studied. Despite these successes, a number of challenges remain. The number of women in care who received no regimen did not decrease substantially over this time period and was still unacceptably high at 7% in 2010; this would represent more than 8000 untreated HIV-infected pregnant Tanzanian women if applied on a national level [13].

Our study had several limitations. First, a longitudinal prospective cohort study would have been a superior design for many reasons. Being a retrospective study on first DBS PCR results only, we were unable to determine transmission rates via breastfeeding, which contributes significantly to pediatric HIV infection in sub-Saharan Africa. Still, inferences about early MTCT in the first several weeks of life are useful. Second, as our cohort only represents those who received an HIV-1 DNA PCR within 75 days of life, it excludes the mother-infant pairs who accessed PMTCT services prior to delivery but were lost before testing. Third, we did not have access to data that may also influence MTCT, such as WHO clinical staging, maternal CD4 count and HIV-1 RNA levels, the latter not being part of routine care. We also did not have the ability to verify time of initiation nor adherence to documented PMTCT regimens for women or their infants. Fourth, data in national registries were occasionally missing or illegible and therefore, data from some individuals were incomplete. Finally, the women in our cohort are women who chose to receive PMTCT services and thus may be more likely to be adherent to medication, both for themselves and their babies, limiting generalizability to the population as a whole and suggesting that overall transmission might actually be higher than our data show.

The retrospective nature of our data collection represents a single point in time for each women and her infant while PMTCT policy, research, and guidelines have changed dramatically over these years. Tanzania is no exception. The changes demonstrated for the time period of 2008–2010 must be taken into context; namely, prior to major changes in the WHO PMTCT guidelines and in particular with the rollout of Option B/B+. Thus, the historical nature of our data does not reflect current practice in these settings. Still, however, the findings have both direct and indirect relevance to current practice. As we demonstrated, there were still women in 2010 who failed to receive any PMTCT services, and women who still received sdNVP only. With changing guidelines, improved access to care, and lower MTCT rates over time, we would expect that MTCT rates in Tanzania have now decreased even further. Today, Tanzania is rolling out option B+ and soon all HIV-infected pregnant women in the country should have access to lifelong HAART, offering the opportunity to decrease perinatal HIV transmissions even further.

## Conclusions

In conclusion, our study demonstrates reductions in MTCT of HIV-1 in three regions of Tanzania coincident with increased use of more effective PMTCT interventions. The country’s change in guidelines in recent years is seen at the field level by the change in treatment regimens over time, an increase in the uptake of maternal prophylaxis, and a subsequent decrease in the use of sdNVP. The use of infant DBS PCR is a valuable tool as an adjunct measurement of the effectiveness of PMTCT regimens and programs. The overall decrease in the proportion of positive PCR over time, as well as the uptake of EID testing, are encouraging for Tanzania.
